# HBV pregenome RNA as a predictor of spontanous HBeAg seroconversion in HBeAg-positive chronic hepatitis B patients

**DOI:** 10.1186/s12876-023-03023-8

**Published:** 2023-11-09

**Authors:** Guangjun Song, Ruifeng Yang, Qian Jin, Juan Liu, Huiying Rao, Bo Feng, Yandi Xie

**Affiliations:** 1 Peking University People’s Hospital, Peking University Hepatology Institute, Beijing Key Laboratory of Hepatitis C and Immunotherapy for Liver Diseases, Peking University, No.11 Xizhimen South Street, Beijing, 100044 China; 2 Research Center for Technologies in Nucleic Acid-Based Diagnostics, Changsha, Hunan China

**Keywords:** Chronic hepatitis B, HBV pgRNA, Spontaneous HBeAg seroconversion

## Abstract

**Background:**

Previous studies have indicated that HBV pregenome RNA (HBV pgRNA) could predict HBeAg seroconversion among the chronic hapatitis B (CHB) patients treated with pegylated interferon (Peg-IFN) or nucleos(t)ide analogues (NAs). However, the data about the prediction of HBV pgRNA for spontaneous HBeAg seroconversion is limited.

**Methods:**

One hundred thirteen CHB patients with HBeAg-positive in the immune active phase were followed up for 76 weeks without antiviral treatment. Based on the laboratory test results of liver function, HBeAg, anti-HBe, and HBV DNA at week 76, patients were assigned to two groups: spontaneous HBeAg seroconversion (group A, *n* = 18) and non-spontaneous HBeAg seroconversion group. Among the latter group, 36 patients were selected as controls (group B, *n* = 36).

**Results:**

At week 12, between group A and group B, there was a significant difference in the level of HBV pgRNA (group A 6.35 ± 1.24 log_10_ copies/ml and group B 7.52 ± 0.79 log_10_ copies/ml, *P* = 0.001), and the difference enlarged at week 28. The receiver operating characteristic curves (AUROCs) of the HBV pgRNA level and the ∆HBV pgRNA at week 28 were 0.912 (*P* = 0.001, 95% CI: 0.830–0.994), and 0.934 (*P* = 0.001, 95% CI: 0.872–0.996), respectively. The optimal cutoffs of HBV pgRNA and the reduction from baseline (∆HBV pgRNA) at week 28 for spontaneous HBeAg seroconversion prediction were 5.63 log_10_ copies/ml and 1.85 log_10_ copies/ml, respectively. The positive predictive value and negative predictive value of HBV pgRNA and ∆HBV pgRNA at week 28 were 86.7% and 87.2%, 87.5% and 89.5%, respectively. And the combination of the HBV pgRNA level and the HBV pgRNA decreased could provide better prediction.

**Conclusions:**

HBV pgRNA is a sound predictor for spontaneous HBeAg seroconversion among the CHB patients in immune active phase. Dynamic monitoring of HBV pgRNA is helpful for clinical treatment decision.

## Introduction

Approximately 240 million persons are chronic HBV surface antigen (HBsAg) carriers with a varying prevalence geographically, highest in Africa and Asia. Deaths from cirrhosis and hepatocellular carcinoma (HCC) worldwide were estimated at 310,000 and 340,000 per year, respectively [1, 2].

According to the natural history of chronic HBV infection, the hallmark of transition from the immune clearance phase to the inactive carrier phase is the loss of serum hepatitis B e antigen (HBeAg) and the development of HBeAg seroconversion. Achieving HBeAg seroconversion with undetectable HBV DNA is a satisfactory endpoint for chronic hepatitis B (CHB) patients with HBeAg‐positive, which represent partial immune control of CHB infection and improved long-term clinical outcomes, including disease remission, a lower incidence of cirrhosis and HCC [3]. The annual rate of spontaneous HBeAg seroconversion is below 2% in children younger than 3 years old and increases during puberty and among adults to 8% and 12%, respectively [4, 5]. Given that HBeAg seroconversion may occur spontaneously, it is of great clinical value to early identify the patients who are prone to spontaneous HBeAg seroconversion, avoiding unnecessary antiviral therapy, potential side effects and financial burden.

Peripheral blood HBV RNA is pregenome RNA(HBV pgRNA), and it is encapsidated and present in HBV-like viral particles in the serum of CHB patients [6]. And it is a direct transcription product of HBV covalently closed circular DNA (cccDNA), so the level might reflect the quantity and the transcriptional activity of intrahepatic cccDNA [7]. In recent years, HBV pgRNA has received increasing interest in monitoring the efficacy of antiviral treatment with pegylated interferon (Peg-IFN) [8–11] or nucleos(t)ide analogues (NAs) [12, 13]. In a cohort, HBeAg-positive patients received 144-week entecavir treatment, the level of HBV pgRNA at week 24 could powerfully predict HBeAg seroconversion with that the patients with the value of HBV RNA less than 5.4log_10_ copies/ml had 72.73% HBeAg seroconversion [14]. However, the data is limited about the prediction of HBV pgRNA for spontaneous HBeAg seroconversion.

In this study, CHB patients with HBeAg-positive were observed without antiviral therapy, and the level of HBV pgRNA was serially quantified to evaluate the value of this new biomarker for the prediction of spontaneous HBeAg seroconversion.

## Materials and methods

This study was approved by the Ethical Committee of Human Experimentation in Peking University People’s Hospital, and was performed in agreement with the Helsinki Declaration of 1975. Patients were enrolled after providing written informed consent.

### Patients and study design

The enrollment of the patients and the study design were seen in the previous study [15]. According to the European Association for the Study of the Liver (EASL) Clinical Practice Guidelines [16], spontaneous HBeAg seroconversion was considered as normal ALT, the HBV DNA level below 2 × 10^3^ IU/ml, and HBeAg negative and anti-HBe presentation. Nonspontaneous HBeAg seroconversion was considered as abnormal ALT, HBeAg positive, or the level of HBV DNA above 2 × 10^3^ IU/ml, or both. Based on the laboratory test results of liver function, HBeAg, anti-HBe, and HBV DNA at week 76, the patients were assigned to two parts: spontaneous HBeAg seroconversion (group A, *n* = 18) and non-spontaneous HBeAg seroconversion (*n* = 95), and 36 patients were selected from the latter part as controls (group B) by simple random sampling. The detail of randomization process was seen in the previous study [17].

### HBV pgRNA Test

At baseline, week 12, week28, week32, week 40, week52, week64 and week76, blood samples were collected and stored at -80℃ until assayed. All samples were quantitatively detected in Sansure Biotech Inc.Changsha, Hunan Province, P. R. China by previously reported methods [9].Briefly, HBV RNA was extracted from 200 μL of serum using a nucleic acid extraction kit (Sansure Biotech Inc. China) which was developed based on the magnetic bead technology [18]. CDNA was synthesized under the temperature of 50 °C for 30 min by reverse transcription from HBV RNA, and the cDNA amplification was performed by an activation step at 95 °C for 2 min, followed by 50 two-step cycles (each cycle 15 s at 95 °C and 30 s at 60 °C), and a cooling step down to 25 °C for 10 s. The fluorescence of cDNA was detected and measured by the 7500 Real-Time PCR System (Applied Biosystems®) with a linear range from1.0 × 10^2^ copies/mL to 1.0 × 10^9^ copies/mL and a lower detection limit of 50 copies/mL.

The methods of biochemical tests, HBsAg, HBeAg and HBV DNA tests have been displayed in the previous study [15, 17].

### Statistical analyses

Chi-square and Student’s t-test were performed up to the data. Logistic regressions were used to assess the odds ratio (OR) and 95% confidence intervals (95% CI) of HBV pgRNA. Receiver operating characteristic curves (ROC) were generated to compare the relative sensitivity and specificity of HBV pgRNA and ∆HBV pgRNA as predictors of spontaneous HBeAg seroconversion. The cutoff value was chosen according to the receiver operating characteristic curve when the sensitivity and specificity were both relatively high. A *P*-value of < 0.05 was considered statistically significant. Statistical analyses were performed using the SPSS version 20.0 (SPSS, Inc., Chicago, IL).

## Results

### Demographics and baseline characteristics

Fifty-four patients were enrolled, and the baseline characteristics of the two groups are summarized in Table 1. Between group A and group B, there were no significant differences in the distributions of gender, age and HBV genotypes, the levels of baseline ALT, HBsAg, HBeAg, HBV DNA and HBV pgRNA.

**Table 1:** Comparison of the two groups of the CHB patients in baseline characteristics

Characteristics	Group A (*n* = 18)	Group B (*n* = 36)	*t*-Test or chi-square test *P* values	Univariate logistic regression *P* values (OR [95% CI])
Age, years (mean ± SD)	24.06 ± 5.60	25.81 ± 5.50	0.28	0.28 (1.07 [0.95, 1.19])
Gender (M/F)	13/5	26/10	0.62	1.00 (1.00 [0.28, 3.54])
ALT (mean ± SD)	187.53 ± 72.78	158.98 ± 60.64	0.13	0.14 (0.99 [0.99, 1.00])
HBV genotype (No. of genotype B/C)	11/7	20/16	0.46	0.70 (0.80 [0.25, 2.52])
HBsAg, log_10_ U/ml (mean ± SD)	4.16 ± 0.52	4.29 ± 0.70	0.50	0.49 (1.36 [0.57, 3.26])
HBeAg, log_10_ PEI-U/ml (mean ± SD)	2.32 ± 0.78	2.60 ± 0.62	0.15	0.15 (1.84 [0.80, 4.24])
HBV DNA, log_10_ IU/ml (mean ± SD)	7.75 ± 0.68	8.05 ± 0.99	0.24	0.24 (1.47 [0.78, 2.78])
HBV pgRNA, log_10_ copies/ml (mean ± SD)	7.68 ± 0.84	7.47 ± 0.92	0.42	0.41 (0.76 [0.39, 1.47])

*SD *Standard deviation, *OR *Odd ratio, *95% CI *95% confifidence interval

### Variation tendency in HBV pgRNA levels

At week 12, between group A and group B, there was a significant difference in the levels of HBV pgRNA (group A 6.35 ± 1.24 log_10_ copies/ml and group B 7.52 ± 0.79 log_10_ copies/ml, *P* = 0.001). And from week 12 to the end of the study, the significant difference in the levels of HBV pgRNA between these two groups persisted.

**Fig. 1:**
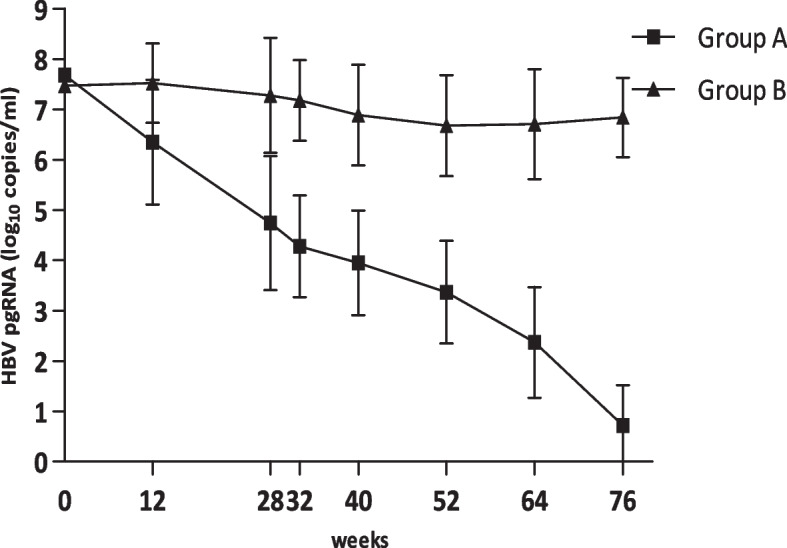
The decline curves of HBV pgRNA levels in group A and B. At week 12, there was significant difference (*P* = 0.001). At weeks 28, 32, 40, 52, 64, and 76, the significance increased

In group A, the levels of HBV pgRNA declined continously, but in group B, no significant decrease was observed. The variation tendencies were displayed in Fig. 1.

### Variable screen for spontaneous HBeAg seroconversion prediction

Variables with *P* < 0.1 in the univariate analysis served as candidate independent variables, including the HBV pgRNA levels at week 12 and 28, the reduction from baseline to week 12 and 28 in HBV pgRNA levels(∆HBV pgRNA). Spontaneous HBeAg seroconversion was considered as the outcome (dependent) variable to be used for multivariate logistic regression analysis with a forward variable selection technique (Table 2).

**Table 2:** Screening variables for the prediction of spontaneous HBeAg Seroconversion

Variables	Group A (*n* = 18)	Group B (*n* = 36)	Student’s *t*-test *P* values	Univariate logistic regression *P* values (OR [95% CI])	Multivariate logistic regression *P* values (OR [95% CI])	AUROC *P* values [95% CI]
HBV pgRNA, log_10_ copies/ml						
12 weeks	6.35 ± 1.24	7.52 ± 0.79	0.001	0.002 (3.82 [1.63, 8.94])	0.028 (4.46 [1.18, 16.85])	0.782 (0.001 [0.646, 0.919])
28 weeks	4.74 ± 1.33	7.28 ± 1.14	0.001	0.001 (3.98 [1.99, 7.94])	/	0.912 (0.001 [0.830, 0.994])
∆HBV pgRNA, log_10_ copies/ml						
12 weeks	1.33 ± 1.30	-0.05 ± 0.89	0.001	0.003 (0.24 [0.09, 0.61])	/	0.816 (0.001 [0.699, 0.934])
28 weeks	2.94 ± 1.38	0.19 ± 1.25	0.001	0.001 (0.22 [0.10, 0.49])	0.001 (0.20 [0.08, 0.54])	0.934 (0.001 [0.872, 0.996])

∆HBV pgRNA, the reduction of the level of HBV pgRNA from baseline; *AUROC* Area under the receiver-operator curve, *OR* Odd ratio, *95% CI* 95% confifidence interval

In the logistic regression equation, only the HBV pgRNA level at week 12 and the ∆HBV pgRNA at week 28 were enrolled, with the odds ratio (OR) of 4.46 and 0.20, respectively [95% confidence interval (CI): 1.18–16.85 and 0.05–0.54, respectively].

And the regression equation was logit(*P*) = -7.687 + 1.494 × HBV pgRNA(week 12) -1.590 × ∆HBV pgRNA(week 28).

### Predictive values of the HBV pgRNA

**Fig. 2:**
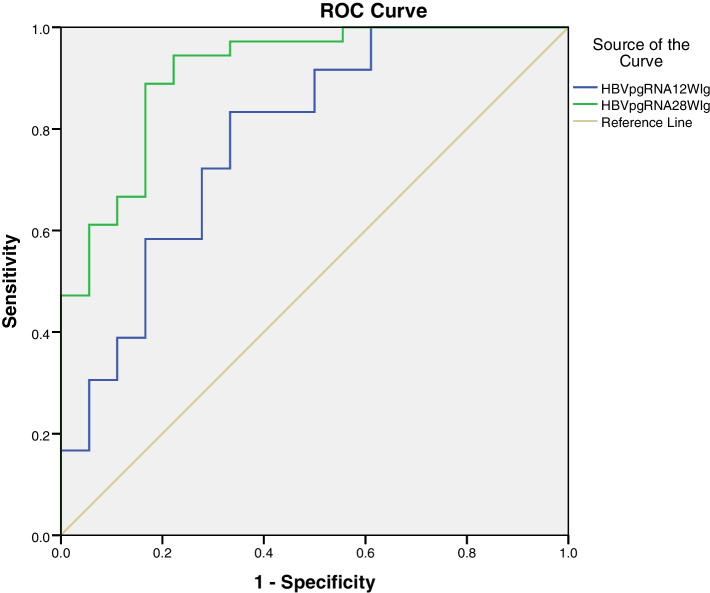
Receiver operating characteristic curves of HBV pgRNA at week 12 and 28, 0.782 and 0.912, respectively

**Fig. 3:**
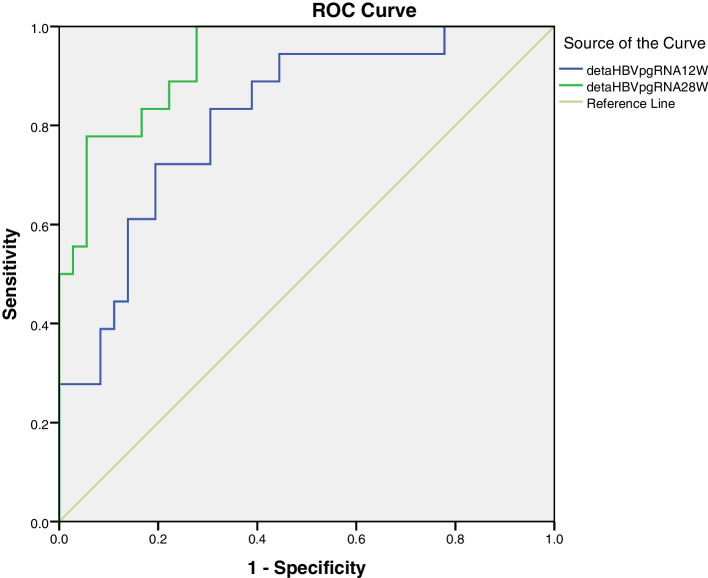
Receiver operating characteristic curves of ∆HBV pgRNA at week 12 and 28, 0.816 and 0.934, respectively

The areas under the receiver operating characteristic curve (AUROC) of the HBV pgRNA levels at week 12 and 28, the ∆HBV pgRNA at week 12 and 28 were shown in Table 2, Figs. 2 and 3. The AUROCs of the HBV pgRNA level and the ∆HBV pgRNA at week 28 were 0.912 (*P* = 0.001, 95% CI: 0.830–0.994), and 0.934 (*P* = 0.001, 95% CI: 0.872–0.996), respectively. And the AUROCs of resluts from the regression equation was 0.941 (*P* = 0.001, 95% CI: 0.876–1.000). The AUROCs were all greater than 0.9, suggesting a satisfactory predictive value.

**Fig. 4:**
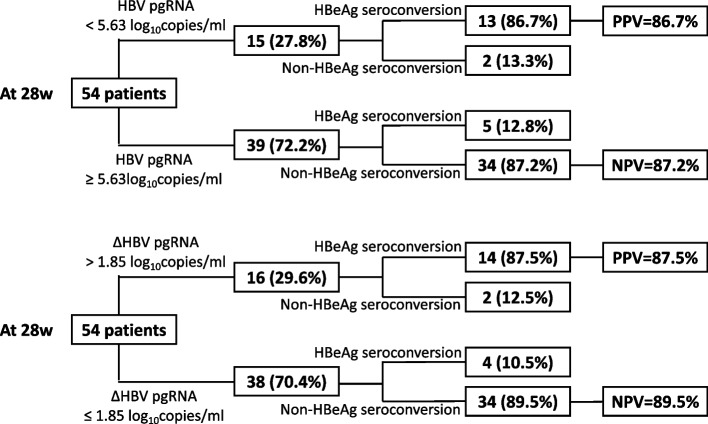
Flowchart of HBV pgRNA and HBV pgRNA decline prediction for spontaneous HBeAg seroconversion

With the corresponding Youden’s index of 0.67 and 0.72, the optimal cutoff values of the HBV pgRNA level at week 28 and the ∆HBV pgRNA at week 28 were 5.63 log_10_ copies/ml and 1.85 log_10_ copies/ml, respectively. The sensitivity, specificity, positive predictive value (PPV), and negative predictive value (NPV) of the optimal cutoff value of the HBV pgRNA level at week 28 were 94.4%, 72.2%, 86.7%, and 87.2%, respectively. The sensitivity, specificity, PPV and NPV of the optimal cutoff value of the ∆HBV pgRNA at week 28 were 77.8%, 94.4%, 87.5%, and 89.5%, respectively (Fig. 4). And the optimal cutoff of the results from the regression equation was -0.831 with the corresponding Youden’s index of 0.778, and the sensitivity, specificity, PPV and NPV were 100%, 77.8%, 100%, and 90%.

## Discussion

According to the inclusion criteria, the chronic hepatitis B patients enrolled in this study were all HBeAg positive and in immune active phase with HBV DNA levels over than 1 × 10^5^ IU/ml and ALT levels ranged from 2 to 10 times the upper limit of normal, and followed up for about one and half years (76 weeks) without antiviral treatment. At the end of follow-up, 18 patients achieved spontaneous HBeAg seroconversion (group A), 36 patients were randomly selected from the other patients without spontaneous HBeAg seroconversion as controls (group B). The serum HBV pgRNA levels declined dramatically in the patients with spontaneous HBeAg seroconversion, and in the patients group B, no significant decline was observed, indicating that serum HBV pgRNA may be a novel predictor of spontanous HBeAg seroconversion in HBeAg-positive chronic hepatitis B patients.

As the transcription template of all HBV transcripts, intrahepatic covalently closed circular DNA (cccDNA) can produce the offspring virion DNA and influence viral proteins synthesis. CccDNA is the molecular basis for the persistence of HBV, maintained in the nucleus as a stable minichromosome [19, 20]. And the eradication of cccDNA is considered to be the gold standard in the clearance of HBV [21]. Therefore, it is of great significance to comprehensively monitor the transcriptional activity of cccDNA for evaluating the efficacy of antiviral therapy and disease progression risk [22]. However, it is not practicable to quantify cccDNA as a routine in real-world clinical practice due to the invasive nature of liver biopsy, inadequate specimen size, the subjectivity of different observers, lack of standardized quantification methods, and potential complications of hepatic puncture [21, 23]. So it is clinically meaningful to explore noninvasive and convenient serum markers indirectly reflecting intrahepatic cccDNA level.

Different from HBsAg derived not only from cccDNA, but also in a profound number from the integrated HBV genome [24] and HBV DNA could be suppressed by nucleos(t)ide analogues (NAs), HBV pgRNA is derived only from intrahepatic cccDNA and not affected by NAs, more accurately reflecting intrahepatic cccDNA transcriptional activity [6, 7]. Thus, serum HBV pgRNA may be a new potential surrogate marker of intrahepatic cccDNA and circumvents the impractical difficulty of measuring intrahepatic cccDNA [25].

Several studies have demonstrated that serum HBV pgRNA was an novel and powerful predictor of HBeAg seroconversion in HBeAg-positive patients treated with pegylated interferon (Peg-IFN) [8–11] or NAs [12–14], but the data on the predictive value of serum HBV pgRNA for spontaneous HBeAg seroconversion is limited.

In this study, the level of serum HBV pgRNA in the group achieving spontaneous HBeAg seroconversion was much lower than in the group not achieving spontaneous HBeAg seroconversion at week 12, and thereafter the differences not only persisted, but also enlarged.

The patients with faster decline in HBV pgRNA levels were more likely to get spontaneous HBeAg seroconversion. Logistic regression analysis indicated that the varibles of the HBV pgRNA levels at week 12 and 28, and the reductions from baseline to week 12 and 28 in HBV pgRNA levels(∆HBV pgRNA) had some predictive value for spontaneous HBeAg seroconversion. The areas under the receiver operating characteristic curve (AUROC) of the HBV pgRNA level and the ∆HBV pgRNA at week 12 were 0.782 and 0.816, respectively, indicating that the predictive values were not satisfactory enough. And the AUROCs of the HBV pgRNA level and the ∆HBV pgRNA at week 28 were 0.912 and 0.934, respectively, suggesting that the the predictive values were much better.

The optimal cutoffs of the HBV pgRNA level and ∆HBV pgRNA at week 28 were 5.63 log_10_ copies/ml and 1.85 log_10_ copies/ml, respectively, with the positive predictive value (PPV) and negative predictive value (NPV) 86.7% and 87.2%, 87.5% and 89.5%, respectively. The CHB patients in immune active phase with the HBV pgRNA level lower than 5.63 log_10_ copies/ml, or with ∆HBV pgRNA greater than 1.85 log_10_ copies/ml at week 28, would likely achieve spontaneous HBeAg seroconversion within 48 weeks, and antiviral therapy would not be needed immediately. On the contrary, The CHB patients in immune active phase with the HBV pgRNA level higher than 5.63 log_10_ copies/ml, or with ∆HBV pgRNA lower than 1.85 log_10_ copies/ml at week 28, are unlikely to get spontaneous HBeAg seroconversion, and antiviral therapy should be initiated as soon as possible. And the combination of the HBV pgRNA level and the HBV pgRNA decreased could provide better prediction.

Compared with previous studies, the predictive value of the HBV pgRNA for spontaneous HBeAg seroconversion was superior to HBV DNA and HBeAg, and similar to HBcrAg. For HBV DNA [15], the PPV and NPV were 81.8% and 91.2%, respectively, but the sensitivity was only 50%. And for HBeAg [15], the NPV with 98.3% was excellent, but the PPV with 30.9% was too low. For HBcrAg [17], the PPV and NPV were about 75% and 95%, respectively, and the sensitivity and specificity were acceptable.

There are still several limitations in this study. The sample size is small, and larger sample studies are expected. All the patients enrolled in this study are Chinese, and the HBV genotype of the patients is B or C. The results of this study need to be further validated in other ethnic populations and patients infected with other genotype of HBV. The duration of follow-up is not long enough, and more time is requried to further observe whether HBeAg reversion will occur.

In conclusion, HBV pgRNA is a sound predictor for spontaneous HBeAg seroconversion among the CHB patients in immune active phase. Dynamic monitoring of HBV pgRNA is helpful for clinical treatment decision.

## Data Availability

The datasets used and/or analysed during the current study are available from the corresponding author on reasonable request.

## Abbreviations

HBV pgRNA: HBV pregenome RNA
CHB: Chronic hapatitis B
HBeAg: Hepatitis B e antigen
PPV: Positive predictive value
NPV: Negative predictive value
